# Oxymatrine Attenuates Osteoclastogenesis via Modulation of ROS-Mediated SREBP2 Signaling and Counteracts Ovariectomy-Induced Osteoporosis

**DOI:** 10.3389/fcell.2021.684007

**Published:** 2021-05-31

**Authors:** Chao Jiang, Qingliang Ma, Shiyu Wang, Yang Shen, An Qin, Shunwu Fan, Zhiwei Jie

**Affiliations:** ^1^Department of Orthopaedics, Sir Run Run Shaw Hospital, Zhejiang University School of Medicine, Hangzhou, China; ^2^Key Laboratory of Musculoskeletal System Degeneration and Regeneration Translational Research of Zhejiang Province, Hangzhou, China; ^3^Department of Orthopaedics, Shanghai Key Laboratory of Orthopaedic Implant, Shanghai Ninth People’s Hospital, Shanghai Jiao Tong University School of Medicine, Shanghai, China

**Keywords:** oxymatrine, osteoclast, ROS, SREBP2, osteoporosis

## Abstract

Osteoporosis, mainly caused by osteoclast-induced bone resorption, has become a major health problem in post-menopausal women and the elderly. Growing evidence indicates that inhibiting osteoclastogenesis is an efficient approach to develop alternative therapeutic agents for treating osteoporosis. In this study, we identified the potential regulating role of Oxymatrine (OMT), a quinazine alkaloid extracted from *Sophora flavescens* with various therapeutic effects in many diseases, on osteoclastogenesis for the first time. We found that OMT attenuated RANKL-induced osteoclast formation in both time- and dose-dependent manners. Further, OMT significantly suppressed RANKL-induced sterol regulatory element-binding protein 2 (SREBP2) activation and the expression of the nuclear factor of activated T cells 1 (NFATc1). Moreover, OMT inhibited the generation of RANKL-induced reactive oxygen species (ROS), and the upregulation of ROS could rescue the inhibition of SREBP2 by OMT. More importantly, ovariectomy (OVX) mouse model showed that OMT could effectively improve ovariectomy (OVX)-induced osteopenia by inhibiting osteoclastogenesis *in vivo*. In conclusion, our data demonstrated that OMT impaired ROS mediated SREBP2 activity and downstream NFATc1 expression during osteoclastogenesis, suppressed OVX-induced osteopenia *in vivo*, which suggested that OMT could be a promising compound for medical treatment against osteoporosis.

## Introduction

Healthy bone homeostasis relies on the balance between osteoblastic bone formation as well as osteoclastic bone resorption. This delicate balance between osteoclast-mediated bone resorption and osteoblast-mediated bone mineralization is disturbed under many conditions, such as malignancies, aging, and estrogen deficiency (Rodan and Martin, 2000). Excessive activation of osteoclasts is considered to be the main cause of estrogen deficiency-induced bone loss (Harvey et al., 2010; Manolagas, 2018; Compston et al., 2019). Thus, it’s imperative to completely elucidate the mechanisms regulating osteoclast differentiation and develop novel agents which could effectively treat osteoporosis.

As multinucleated giant cells participating in bone homeostasis, osteoclasts are differentiated from hematopoietic progenitors under macrophage colony-stimulating factor (M-CSF) and cytokine receptor activator of nuclear factor-κB ligand (RANKL) stimulations (Gingery et al., 2003; Boyce and Xing, 2007; Xu and Teitelbaum, 2013). RANKL recruits TNF receptor-associated factor 6 (TRAF6) via interacting with its receptor RANK, which further activates downstream effectors such as nuclear factor-κB and mitogen-activated protein kinases (MAPKs), including extracellular signal-regulated kinase (ERK)1/2, p38 MAPK, and c-jun N-terminal kinase (JNK), which ultimately resulting in the activation of activator protein-1 and NFATc1 (Asagiri et al., 2005; Takayanagi, 2007; Yamashita et al., 2007).

NFATc1 is a terminal regulator that promotes osteoclast differentiation, and directly affects osteoclast differentiation and expression of osteoclast-related genes, such as tartrate-resistant alkaline phosphatase (TRAP), cathepsin K (CTSK) and dendritic cell-specific transmembrane protein, which promote osteoclast differentiation and function (Takayanagi, 2007; Negishi-Koga and Takayanagi, 2009; Boyce, 2013). Previous study has reported that NFATc1-deficiency in osteoclast precursor cells cannot differentiate into osteoclasts, and NFATc1-knockout mice exhibits a serious osteopetrosis because of the increase of bone mass (Winslow et al., 2006). Multiple signaling pathways are reported to be connected with NFAT activation. The activation of NF-κB and c-Fos signaling pathways up-regulates NFATc1 expression by RANKL stimulation (Franzoso et al., 1997; Neale Weitzmann and Pacifici, 2005; Boyce et al., 2015). Moreover, NFATc1 was reported to be auto-amplified during osteoclastogenesis (Asagiri et al., 2005). Sterol regulatory element-binding protein 2 (SREBP2) is a vital transcription factor which regulates cholesterol synthesis (Steen et al., 2017). In our previous study, we found that SREBP2 could transcriptionally regulate NFATc1 expression through binding to SRE of the NFATc1 promoter (Jie et al., 2019). However, the underlying mechanism of RANKL induced SREBP2 activation remains ill-defined.

Numerous studies indicates that the accumulation of intracellular reactive oxygen species (ROS) is a vital regulator related to the osteoclast-associated disease occurrences (Manolagas, 2010; Callaway and Jiang, 2015; Agidigbi and Kim, 2019). RANKL-induced ROS further stimulate osteoclastogenesis by activating downstream signaling pathways such as NF-κb and MAPK, which are important for osteoclastogenesis (Lean et al., 2003; Lee et al., 2005; Jung et al., 2008). These researches might provide evidence for the view that reducing ROS is a promising strategy for treating osteoporosis.

Oxymatrine (OMT) is a quinazine alkaloid extracted from *Sophora flavescens* which has been reported to exhibit various therapeutic effects, such as immunomodulation, anti-inflammation, anti-oxidation, anti-tumor, anti-virus, and hepatoprotection (Lean et al., 2003; Lee et al., 2005; Jung et al., 2008; Huang et al., 2020; Li et al., 2020a, b; Wang et al., 2020; Xiang et al., 2020). However, there is no report on the treatment of osteoporosis with OMT. In the present study, we conducted *in vitro* experiments to test the effect of OMT on osteoclasts differentiation, activity and its underlying mechanisms. An ovariectomy (OVX) induced osteoporosis model was also used to clarify the potential therapeutic effects of OMT.

## Materials and Methods

### Materials and Main Reagents

Oxymatrine was obtained from Selleck (Houston, Texas, United States). DMSO were obtained from Sigma-Aldrich (St. Louis, MO, United States). Recombinant soluble mouse M-CSF and RANKL were purchased from R&D Systems (Minneapolis, MN, United States). DMEM (Dulbecco’s modified eagle medium), α-MEM (Eagle’s minimal essential medium with Alpha Modification), fetal bovine serum (FBS) as well as penicillin/streptomycin were all from Gibco-BRL. To prevent photosensitivity, the assays were all performed in the absence of visible light. To make DMSO content < 0.1%, OMT was diluted in cell culture medium, meanwhile, same concentration of DMSO used as a control group. Antibodies for ERK (extracellular signal-regulated kinase) (#4695), p38 (#8690), JNK (c-Jun N-terminal kinase) (#9252), phosphorylated p-ERK (Thr202/Tyr204) (#4370), p-p38 (Thr180/Tyr182) (#4511), p-JNK (Thr183/Tyr185) (#4668), NFATc1 (#8032) and GAPDH (#51332) were purchased from Cell Signaling Technology. TRAP (ab191406), Cathepsin K (ab187647), SREBP2 (ab30682) and NOX1 (ab131088) were purchased from Abcam. TRAP staining kit were obtained from Sigma-Aldrich.

### Cell Culture and Transfection

The isolation of primary bone marrow-derived macrophages (BMMs) from the entire bone marrow of male 8-week-old C57BL/6 mice were done as previously reported (Chen et al., 2015). Briefly, cells from the bone marrow of femurs and tibias were obtained, and cultured in α-MEM with 1% penicillin/streptomycin, 10% FBS, and 30 ng/mL M-CSF in an 37°C incubator with 5% CO_2_. RAW264.7 cells were cultured in DMEM with 10% FBS. The culture medium was changed every 2 days. Mouse Flag-SREBP2 plasmids were purchased from GeneChem (Shanghai, China). Transfections were performed using Lipofectamine 3000 (Invitrogen) according to the manufacturer’s instructions.

### CCK-8 Assay

To evaluate the potential cytotoxicity of OMT on BMMs, CCK-8 assay was performed. Cells were cultured with a series of concentrations of OMT (0, 25, 50, 100, 200, 400, and 800 μM) for 48 as well as 96 h in 96-well plates. After treatment, the medium was changed with serum-free medium containing 10% CCK-8 solution and cells were incubated for another 2 h. The optical density (OD) at 450 nm was then detected to calculate relative cell viability.

### Flow Cytometry for Apoptosis

BMMs were seeded in 6-well plates and incubated with 0, 100, 200 and 400 μM OMT for 2 days. Cell were then stained with Annexin V-FITC and PI for 15 min at room temperature in a dark room. Using a FACS Canto II (BD), signals of 10,000 cells were detected at 585/42 (564–606 nm) and 702/64 (670–35 nm). Both early and late apoptotic populations were calculated and included as apoptotic cells.

### In vitro Osteoclastogenesis

BMMs were cultured in 96-well plates (8 × 103 cells per well), in quadruplicate. The culture medium was changed every other day. Cells were cultured for 5 days with 30 ng/mL M-CSF and 50 ng/mL RANKL. Then cells were washed 3 times with PBS, fixed for 20 min with 4% paraformaldehyde, and stained with TRAP staining kit. Osteoclasts were considered as TRAP-positive cells which develops over 5 nuclei.

### Bone Resorption Assay

Bovine bone slices were used for bone resorption assays. BMMs were seeded onto bovine bone slices in 96-well plates and then cultured with 30 ng/mL M-CSF and 50 ng/mL RANKL for 5 days to obtain mature osteoclasts. Then cells were treated with 0, 100, 200, and 400 μM OMT for another 5 days. The slices were taken out and the cells were removed from the surface of slices. The osteoclast activity was evaluated by resorption pits which was captured by a scanning electron microscope (FEI Instruments, Hillsboro, OR, United States).

### Quantitative Real-Time PCR

After washed 2 times by PBS, cells were lysed with TRIzol reagent (Invitrogen, Carlsbad, CA). Extracted RNA was reverse-transcribed for cDNA, which was used as templates for quantitative real-time PCR (qRT-PCR) using an ABI Prism 7500 system (Applied Biosystems, Foster City, CA). The cycling conditions were set as previously described: 95°C for 10 min, followed by 35 cycles at 95°C for 15 s and 60°C for 1 min (Xie et al., 2018). The reaction system was 10 μL UltraSYBR Mixture (CWBIO, Beijing, China), 2 μL cDNA, 6 μL ddH_2_O, and 1 μL forward as well as reverse primers. The primer sequences were listed in Supplementary Table 1. The value was normalized to the *Gapdh*. The 2-ΔΔCt method were used to calculate the fold change expression of genes.

### Western Blot Analysis

RIPA lysis buffer was used to extract total protein from cells. After centrifuged the lysates, supernatants were obtained and quantified. Proteins were separated by 10% SDS-PAGE. Then they were transferred to polyvinylidene difluoride (PVDF) membranes. 5% non-fat dry milk dissolved in TBST was used to block the membranes for 1 h at room temperature. Then the membranes were incubated with specific primary antibodies at 4°C overnight. A LAS-4,000 Science Imaging System (Fujifilm, Tokyo, Japan) was used to detect protein bands and obtain images. The Image J software was used for analysis.

### Chromatin Immunoprecipitation (ChIP) Assays

A Chromatin IP kit (Cell Signaling Technology #9002) was used for cell extraction preparation according to the manufacturer’s protocol, as described previously (Jie et al., 2019). Briefly, after OMT treatment followed by RANKL for 48 h, BMM cells were treated with 1% formaldehyde for 10 min to crosslink chromatin and protein, collected and digested to produce chromatin fragments for incubation with IgG or specific antibodies for SREBP2 respectively. Protein A/G agarose beads were used to incubate the immuoprecipitates. After washed several times, the protein–DNA complex was reversed. Finally, ChIP DNA was amplified and analyzed using qPCR.

### Osteoblastogenesis Experiments *in vitro*

MC3T3-E1 cells were obtained from American Type Culture Collection (ATCC; Manassas, United States) and cultured in α-MEM. To perform *in vitro* osteoblast differentiation, 1 mM β-glycerophosphate, and 5 mM L-ascorbic acid 2-phosphate in α-MEM was used as osteogenic medium. For ALP staining, MC3T3-E1 cells were cultured with osteogenic medium and different doses of OMT (0, 100, 200, 400 μM) for 7 days. Afterward, cells were fixed with 4% paraformaldehyde and stained for ALP according to manufacturer’s protocols. For ARS staining, cells were cultured as indicated above for 21 days. After fixation, cells were incubated with 1% Alizarin Red S solution for 5 min at 37°C. All the images were taken using an optical microscope containing a digital camera.

### Animal Model

The animal models were established as described previously (Chen et al., 2015). OMT (10 mg/kg) was intraperitoneal injected every 2 days for 4 weeks before sacrifice. The left tibiae from all the mice were isolated and analyzed with a high-resolution micro-CT. The right tibiae were isolated for histologic or immunohistochemical analysis.

### Micro-CT Scanning

The tibiae were fixed and then analyzed with a high-resolution μCT (Skyscan 1072) instrument. The scanning parameters was set as previously described (Xie et al., 2018). An isometric resolution of 9 μm, with X-ray energy settings of 70 kV and 80 μA. We selected 0.5 mm from the tibia growth plate for further qualitative and quantitative analysis. Each sample we examined BV/TV (trabecular bone volume per total volume), mean Tb.Th (trabecular thickness), mean Tb.N (trabecular number), and mean Tb.Sp (trabecular separation) as reported previously.

### Bone Histomorphometry, Immunohistochemical Analysis, and Calcein Bone Labeling

Histomorphometry analysis was done as previously described (Xie et al., 2018). The hind limb was decalcified in 10% EDTA for 28 days. Decalcified bones were paraffin-embedded and sectioned. Histological sections were prepared for different assays including TRAP, H&E (hematoxylin and eosin), and immunohistochemical experiments, according to the manufacturer’s protocol.

For calcein bone labeling, 15 mg/kg calcein solution was intraperitoneal injected twice on the 10th day and 3rd day before sacrifice. The tibias were separated and sectioned. The calcein labels were captured to calculate mineral apposition rates (MAR).

### ELISA

Blood samples were collected from each mouse and centrifugated at 3,000 rpm for 10 min to obtain serum. Serum concentrations of C-terminal telopeptide of type I collagen (CTx-1), tartrate resistant acid phosphatase 5 (Acp5) and procollagen I N-terminal peptide (P1NP) were examined by ELISA kits (Cusabio, Wuhan, China), according to the manufacture’s instruction.

### Statistical Analysis

The experiments were repeated independently at least 3 times in this work. Data are displayed as mean ± standard deviation (SD). Prism 6 (GraphPad Software, Inc., San Diego, CA, United States) were used for statistical analyses. Student’s *t*-test and one-way ANOVA followed by Tukey’s *post hoc* analysis when appropriate were used to determine statistical differences. ^∗^*P* < 0.05, ^∗∗^*P* < 0.01, ^∗∗∗^*P* < 0.001.

## Results

### OMT Inhibits RANKL-Induced Osteoclast Formation and Activity *in vitro*

**FIGURE 1:**
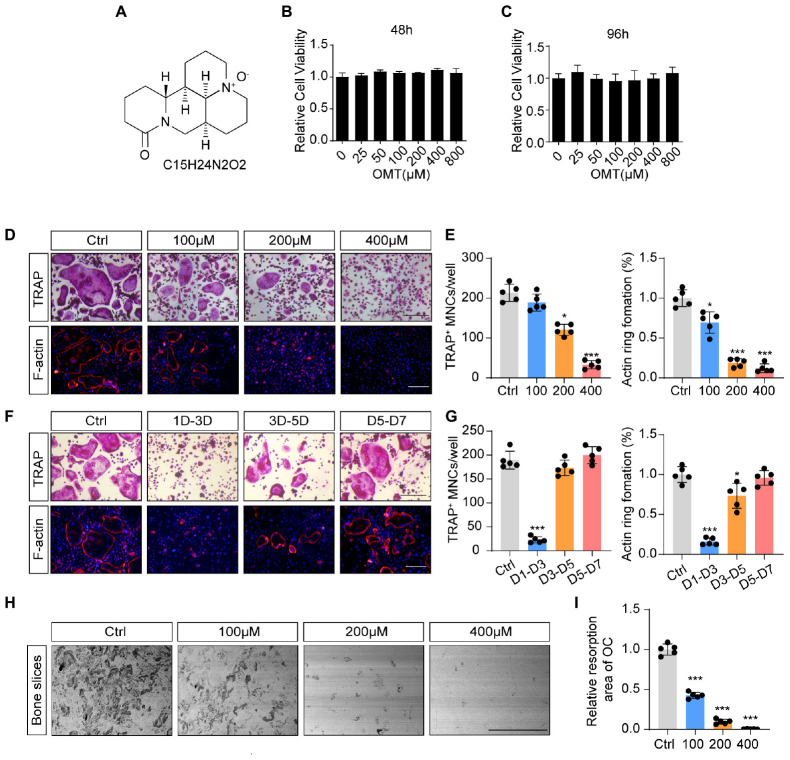
OMT inhibits RANKL-induced osteoclast formation and activity *in vitro*. **(A)** The chemical structure and formula of OMT. **(B,C)** Cell viability of OMT-treated BMMs at 48 and 96 h. **(D)** BMMs were stimulated by 30 ng/mL M-CSF and 50 ng/mL RANKL, and treated with indicated concentrations of OMT for 5 days. Representative images of TRAP staining and F-actin staining were shown. Scale bar = 200μm. **(E)** Quantification of TRAP-positive multinuclear cells and F-actin ring formation rate. **(F)** BMMs were stimulated with 30 ng/mL M-CSF and 50 ng/mL RANKL for 7 days, and treated with 200 μM OMT for the indicated days. TRAP staining and F-actin ring staining were performed. Scale bar = 200μm. **(G)** Quantification of TRAP-positive multinuclear cells and F-actin ring formation rate. **(H)** Representative images of bone resorption pits. Scale bar = 500 μm. **(I)** Quantification of resorption pit area in each group. Data were presented as means ± SD of 5 independent experiments. *p < 0.05, ***p < 0.001.

The chemical structure and formula of OMT are displayed in Figure 1A. To evaluate the potential cytotoxicity of OMT on BMMs, CCK-8 assay was performed to assess cell viability. The results in Figures 1B,C suggested that there was no cytotoxicity affecting BMM survival at the concentration used in this study. In addition, treatment with OMT did not affect the cell apoptosis rate of BMMs (Supplementary Figure 1). Numerous TRAP + multinucleated osteoclasts and F-actin ring formation were observed in control group but significantly inhibited by OMT in a dose-dependent manner, especially at the concentration of 400 μM. (Figures 1D,E). And by treating with 200 μM OMT at indicated time phases during osteoclast formation, we found that OMT mainly exerted its inhibitory effect during early stage (days 1–3) of osteoclast differentiation, rather than mid-late stage (days 3–7) (Figures 1F,G). Moreover, bone resorption assay indicated that OMT inhibited the resorptive activity of osteoclasts dose dependently (Figures 1H,I). These results suggested that OMT could inhibit osteoclastogenesis by impairing early stage of osteoclast differentiation without inducing cytotoxicity.

### OMT Inhibits Osteoclast-Specific Genes Expression

**FIGURE 2:**
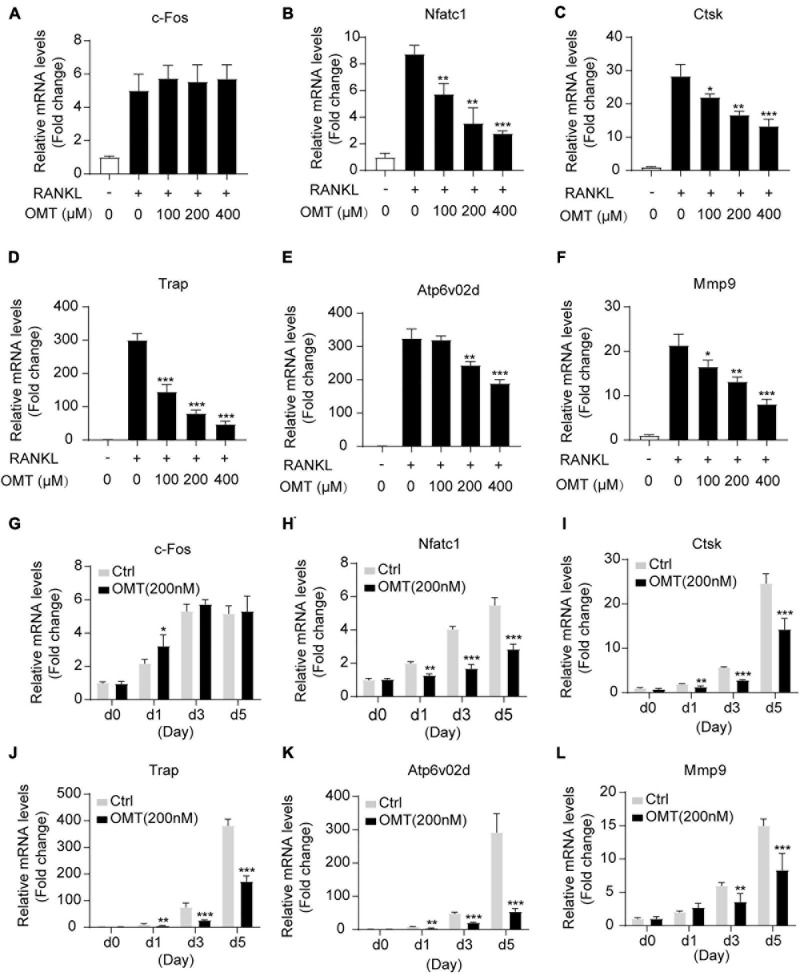
OMT inhibits RANKL-induced osteoclast-specific gene expression *in vitro*. **(A–F)** BMMs were treated with M-CSF, RANKL and indicated concentrations of OMT for 5 days, the expression of *c-fos, Nfatc1, Ctsk, Trap, Atp6v02d*, and *Mmp9* were analyzed by real-time PCR and normalized relative to *Gapdh*. **(G–L)** BMMs were treated with M-CSF, RANKL, and 200 μM OMT for 0, 1, 3, 5 days, the expression of osteoclast-specific genes was tested. Data were presented as means ± SD of 5 independent experiments. *p < 0.05, **p < 0.01, ***p < 0.001.

To further assess the inhibitory effects of OMT on osteoclastogenesis, we then investigated the effects of OMT on the expression of RANKL-induced osteoclast-related genes, including *c-fos, Nfatc1, Ctsk, Trap, Atp6v0d2*, and *Mmp9*. As shown in Figures 2A–F, *Nfatc1, Ctsk, Trap, Atp6v0d2*, and *Mmp9* genes were up-regulated by RANKL, and at the same time, their expression was inhibited by OMT treatment in a dose-dependent manner. However, there was no effect on the expression of *c-fos*. Moreover, we observed the expression of *Nfatc1, Ctsk, Trap, Atp6v0d2*, and *Mmp9* genes was suppressed when cells was treated with OMT during RANKL-induced osteoclastogenesis (Figures 2G–L). Taken together, OMT could down-regulate the mRNA expression of most osteoclast-specific genes except *c-fos.*

### OMT Impaires SREBP2 Activity and Downstream NFATc1 Expression During Osteoclastogenesis

**FIGURE 3:**
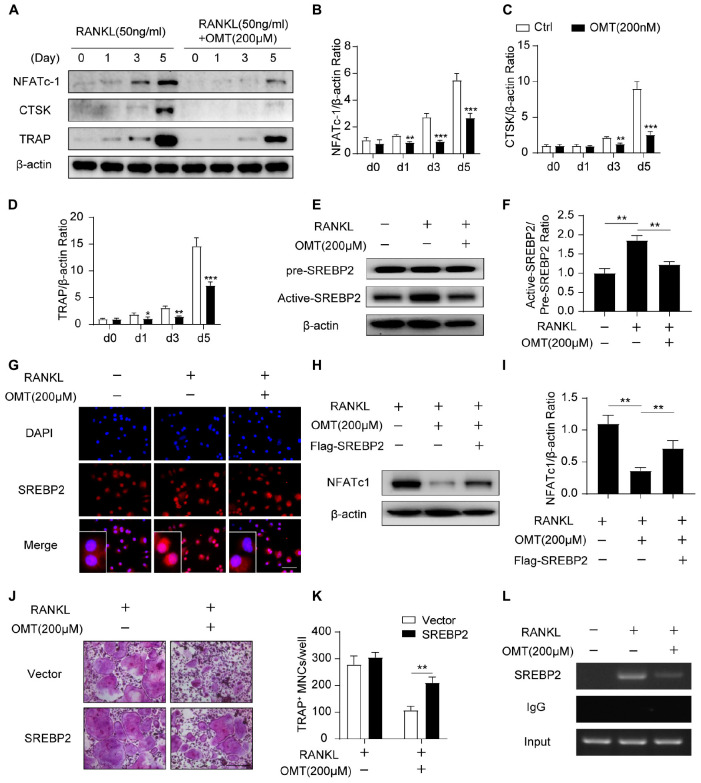
OMT attenuates SREBP2 activity and downstream NFATc1 expression during osteoclastogenesis. **(A)** BMMs were stimulated with RANKL, with or without 200 μM OMT for 0, 1, 3, 5 days, the expression of NFATc1, CTSK and TRAP was tested by western blots. **(B–D)** Quantification of the ratios of band intensity of NFATc1, TRAP, and CTSK relative to β-actin (*n* = 3 per group). **(E)** BMMs were treated with RANKL and 200 μM OMT as indicated, western blot was used to detect the level of pre-SREBP2 and active-SREBP2. **(F)** Quantification of active-SREBP2/pre-SREBP2 ratio (*n* = 3 per group). **(G)** RAW264.7 cells were treated with RANKL and OMT as indicated, immunofluorescence assay was performed to detect SREBP2 translocation. Scale bar = 100 μm. **(H)** BMMs were transfected with Flag-SREBP2 plasmid or empty vector, then treated with RANKL and OMT as indicated, the expression of NFATc1 was examined. **(I)** Quantification of NFATc1/β-actin ratio (*n* = 3 per group). **(J)** BMMs were transfected with Flag-SREBP2 plasmid or empty vector, then treated with RANKL and OMT as indicated. Representative images of TRAP staining were shown. Scale bar = 200 μm. **(K)** Quantification of TRAP-positive multinuclear cells per well (*n* = 5 per group). **(L)** ChIP assay was performed on BMMs, treated with RANKL and OMT as indicated. Data were presented as means ± SD. *p < 0.05, **p < 0.01, ***p < 0.001.

NFATc1 is the major transcriptional regulator during osteoclastogenesis (Asagiri et al., 2005). In the present study, Western blot (Figure 3A) results demonstrated that OMT significantly attenuated RANKL induced up-regulated expression of NFATc1 (Figure 3B). In addition, OMT abrogates the elevation of CTSK (Figure 3C) and TRAP (Figure 3D), which are regulated by NFATc1 and needed for osteoclast formation and function. To further elucidate the potential mechanism by which OMT attenuates NFATc1 expression during osteoclastogenesis, we next analyzed the protein levels of Active-SREBP2 (muture) and pre-SREBP2 (precursor). Interestingly, OMT attenuated RANKL-stimulated activating SREBP2 (Figures 3E,F). Further immunofluorescence analysis exhibited that OMT could inhibit SREBP2 nuclear translocation (Figure 3G).

Next, we treated BMMs with OMT following transfected with Flag-SREBP2 plasmid or empty vector. As shown in Figure 3H, western blot analysis demonstrated that NFATc1 expression was partially rescued (Figures 3,I). Consistent with this, osteoclast formation was inhibited in cells treated with OMT, whereas the impaired osteoclastogenesis was partially rescued by SREBP2 overexpression (Figures 3J,K). There results suggested that SREBP2 overexpression rescued the inhibitory effect of OMT on osteoclast formation. Moreover, we predicted three potential SREBP2 binding sites in the promoter region of NFATc1, according to the JASPAR database. Furthermore, we used a chromosome immunoprecipitation (ChIP) assay and indicated that OMT reduced the binding capacity of SREBP2 on the promoter region of NFATc1 (Figure 3L). Collectively, these results suggest that OMT modulated SREBP2 activation, further affecting NFATc1 expression and inhibiting osteoclast formation.

### OMT Inhibits SREBP2 Activation via Down-Regulating ROS Levels During Osteoclastogenesis

**FIGURE 4:**
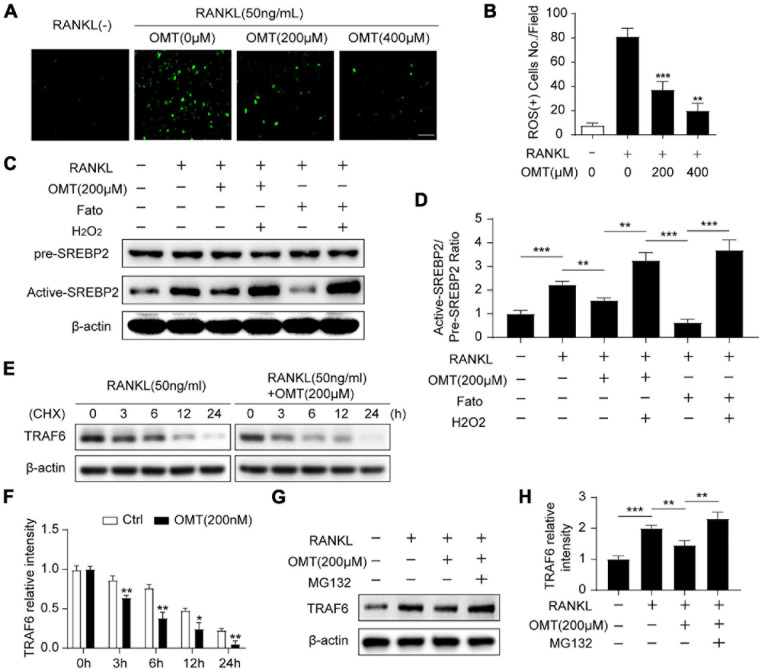
OMT inhibits SREBP2 activation via down-regulating ROS levels during osteoclastogenesis. **(A)** BMMs were treated with RANKL and different concentrations of OMT for 3 days, DCFH-DA was used to examine the intracellular ROS levels and representative images were shown. Scale bar = 100 μm. **(B)** Quantification of ROS-positive cell numbers per field (*n* = 5 per group). **(C)** BMMs were treated with RANKL, OMT, Fatostatin (Fato), and H_2_O_2_ as indicated, the level of pre-SREBP2 and active-SREBP2 was detected with western blots. **(D)** Quantification of the ratios of band intensity of active-SREBP2 relative to pre-SREBP2 (*n* = 3 per group). **(E)** BMMs were treated with or without 200 μM OMT in the presence of RANKL, and cultured with CHX for the indicated hours, the level of TRAF6 was examined by western blots. **(F)** Quantification of the ratios of band intensity of TRAF6 relative to β-actin (*n* = 3 per group). **(G)** TRAF6 expression in BMMs incubated with the proteasomal inhibitor MG 132 in the presence or absence of RANKL and OMT. **(H)** Quantification of the ratios of band intensity of TRAF6 relative to β-actin (*n* = 3 per group). Data were presented as means ± SD. *p < 0.05, **p < 0.01, ***p < 0.001.

We next investigated whether OMT regulates ROS levels during osteoclastogenesis. The intracellular production of ROS was measured using a fluorogenic dye (DCFDA) (Guo et al., 2020b). As shown in Figure 4A, RANKL stimulation significantly increased intracellular ROS, whereas the ROS levels was significantly lowered by OMT treatment (Figure 4B). Moreover, western blot analysis demonstrated that SREBP2 activation was promoted by RANKL, which was down-regulated by OMT and a specific SREBP2 inhibitor Fatostatin. Meanwhile, the ROS agonist H_2_O_2_ could rescue the inactivation of SREBP2 (Figures 4C,D). These results suggest that OMT inhibits RANKL-induced osteoclast differentiation at least in part through suppressing ROS-dependent SREBP2 activation.

TRAF6 functions as a key upstream activator of Nox1-induced ROS signaling in RANKL-induced osteoclastogenesis (Lee et al., 2005). Therefore, we furtherly explore the effect of OMT on TRAF6. Western blot analysis showed that RANKL stimulated proteins level of TRAF6 and Nox1 could be inhibited by OMT in a dose-dependent manner (Supplementary Figure 2), which indicated TRAF6/Nox1 pathway was involved in OMT mediated reduced ROS production. Moreover, TRAF6 protein was degraded more rapidly by OMT treatment than control group, implying OMT regulates TRAF6 protein degradation (Figures 4E,F). However, in the presence of proteasome inhibitor MG132, the degradation of TRAF6 by OMT treatment was suppressed (Figures 4G,H). Furthermore, western blot assays showed that OMT inhibited both NF-κB and MAPK pathways (Supplementary Figures 3A,B), which were considered as downstream signaling pathways of TRAF6 during osteoclast differentiation. Collectively, these results indicated that OMT inhibited ROS production by promoting proteasomal degradation of TRAF6.

### OMT Has no Effect on Osteoblast Differentiation and Osteogenic Gene Expression

**FIGURE 5:**
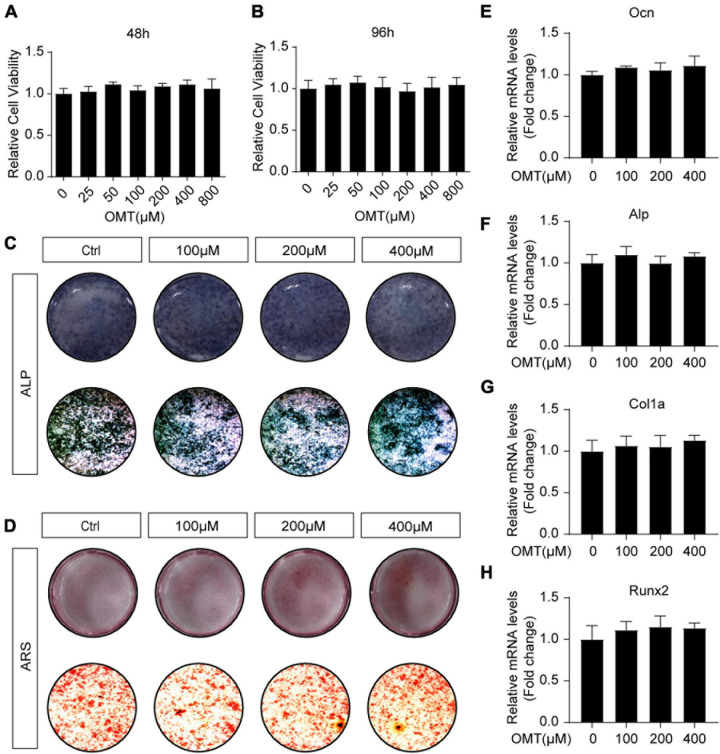
OMT makes no difference to osteoblast differentiation and osteogenic gene expression. **(A,B)** Cell viability of OMT-treated MC3T3-E1 cells at 48 and 96 h. **(C)** MC3T3-E1 cells were treated with different concentrations of OMT for 7 days, representative images of ALP staining were shown. **(D)** MC3T3-E1 cells were treated with different concentrations of OMT for 21 days, representative images of Alizarin Red S staining were shown. **(E–H)** The expression of *Ocn, Alp, Col1a, and Runx2* was tested by real-time PCR after MC3T3-E1 cells were treated with OMT for 7 days. Data were presented as means ± SD of 5 independent experiments.

The effects of OMT on osteoblast differentiation was evaluated. MC3T3-E1 cells were treated with OMT (0–800 μM) for 48 and 96 h, and cell viability was determined with the CCK-8 assay (Figures 5A,B). Interestingly, alkaline phosphatase (ALP) staining and Alizarin Red S staining of MC3T3-E1 cells showed that OMT did not affect the differentiation and mineralization of osteoblasts (Figures 5C,D). Moreover, OMT has no inhibitory role in the expression of osteoblast related genes, including Ocn, Alp, Col1a, and Runx2 (Figures 5E–H). There results suggested that OMT didn’t affect the differentiation and mineralization of osteoblast.

### OMT Improves OVX-Induced Bone Loss by Inhibiting Osteoclast Activity *in vivo*

**FIGURE 6:**
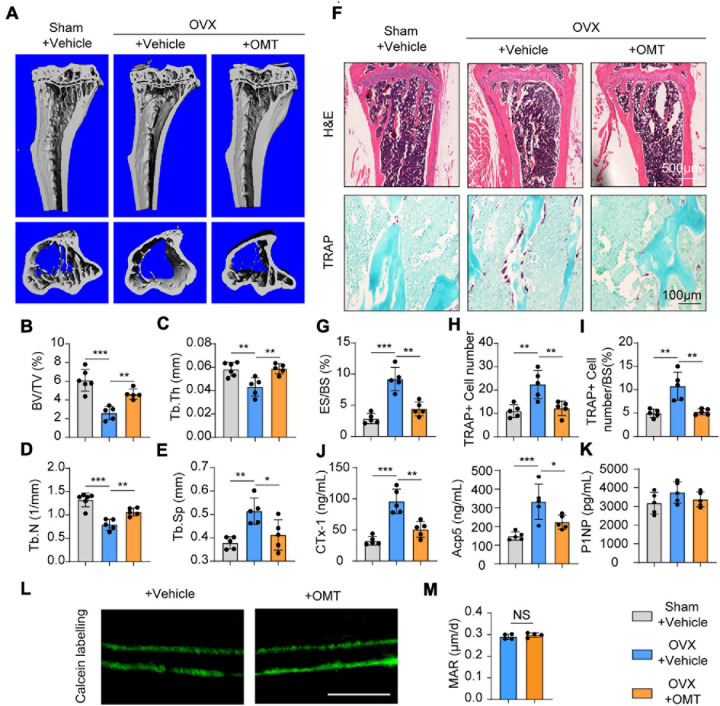
OMT ameliorates OVX-induced bone loss by inhibiting osteoclast formation and activity. **(A)** Representative micro-CT images of tibias from Sham + vehicle, OVX + vehicle and OVX + 10 mg/kg OMT groups. **(B–E)** Quantitative analyses of parameters regarding bone microstructure, including BV/TV, Tb.Th, Tb.N, and Tb.Sp. **(F)** Representative images of H&E staining and TRAP staining from three groups were shown. **(G–I)** Quantitative analyses of the percentage of eroded surface/bone surface (ES/BS), TRAP-positive cell number, and TRAP-positive cell number/bone surface. **(J)** Serum concentrations of CTx-1 and Acp5 from different groups. **(K)** Serum concentrations of P1NP from different groups. **(L)** Representative images of calcein labeling from the vehicle and OMT groups. Scale bar = 5μm. **(M)** Quantification of mineral apposition rates (MAR). Data were presented as means ± SD of 5 independent experiments. *p < 0.05, **p < 0.01, ***p < 0.001.

Based on the data above, we performed ovariectomy on mice as an animal model of postmenopausal osteoporosis to test the effects of OMT on osteoporosis. No toxicity was found in the OMT-treated group (Supplementary Figure 4). The results of micro-CT analyses showed that trabecular bone was significantly reduced in OVX mice compared with that in sham-operated mice, whereas OMT treatment groups decreased bone loss (Figure 6A). Further analysis found that OMT treatment groups display an increased trabecular BV/TV ratio trabecular thickness and number, but reduced trabecular separation compared to OVX mice (Figures 6B–E). Similarly, H&E staining results suggested higher bone mass in OMT treatment groups than that in the OVX mice (Figures 6F,G). Moreover, the results of TRAP staining showed that much more mature TRAP-positive osteoclasts were present in the OVX mice and lower numbers of stained osteoclasts were observed in the OMT treatment groups (Figures 6H,I). Further, ELISA results demonstrated that the CTx-1 and Acp5 protein levels were significantly reduced in the OMT treatment group compared with the OVX mice (Figure 6J), while no significant changes of the bone formation marker P1NP were found in the serum samples (Figure 6K). Calcein labeling also demonstrated that OMT didn’t affect the MAR *in vivo* (Figures 6L,M). Altogether, these results indicated OMT could inhibit osteoclast activity *in vivo* and therefore ameliorate OVX-induced bone loss without affecting bone formation.

## Discussion

As one of the important cells in bone metabolism, osteoclasts play an important role in the development of many skeletal diseases such as osteoporosis, osteoarthritis, cancer induced osteolysis and so on (Rodan and Martin, 2000; Manolagas, 2018). Abnormal activation of osteoclasts in postmenopausal women with osteoporosis leads to a series of bone-related events that seriously affect the quality of life (Compston et al., 2019). People hope that the mechanism of osteoclast differentiation and resorption function will be used to find suitable therapeutic targets to relieve the suffering of patients. In fact, more and more drugs have been studied to play a therapeutic role in osteoporosis (Rachner et al., 2011; Black et al., 2012). Nevertheless, due to the complexity of different diseases and the serious adverse reactions caused by certain drugs, drug researches on osteoclasts have never stopped.

Our previous study has provided evidence that SREBP2 is an important transcription factor which regulates osteoclast formation and activity, breast cancer cell migration and invasion (Jie et al., 2019). It has been reported that the binding of RANKL to its receptor RANK activates the transcription activity of SREBP2 and increases the content of cholesterol, which promotes osteoclast differentiation (Zheng et al., 2020). Therefore, SREBP2 plays an important role in the development of osteoporosis and serves as a potential target. In this work, we found for the first time that OMT, a natural compound extracted from *Sophora flavescens*, could attenuate osteoclastogenesis via inhibiting the activation of SREBP2.

NFATc1 functions as a key transcription factor of osteoclast differentiation which promotes the expression of osteoclast-specific genes (Takayanagi, 2007; Negishi-Koga and Takayanagi, 2009; Boyce, 2013). The NFATc1 deficient mice exhibits impaired osteoclastogenesis and severe osteopetrosis (Asagiri et al., 2005). In this study, OMT treatment decreased the expression of NFATc1. Although the reduction could be caused by the inhibition of MAPK pathway and NF-κB signaling, our data demonstrated a potential role of SREBP2 in this process. The activation of SREBP2 could directly promote the expression of NFATc1 and benefit for osteoclastogenesis, which was blocked by the inactivation of SREBP2 induced by OMT. Therefore, SREBP2 is one of the upstream regulators of NFATc1.

Studies have focused on the downstream signaling of SREBP2 during osteoclastogenesis (Jie et al., 2019; Guo et al., 2020a; Zheng et al., 2020), however, the underlying mechanism by which RANKL regulates SREBP2 activation remains unclear. Kyuhwa Seo found that ROS activate SREBP2, which induces Lipin1 expression in Huh7 and AML12 cells (Seo and Shin, 2017). Since ROS generation is mediated by RANKL and plays an important role in osteoclastogenesis, we hypothesized that the RANKL-induced activation of SREBP2 is mediated by ROS. Indeed, we found that OMT inhibited RANKL-mediated ROS production, accompanied by impaired activation of SREBP2. Consistent with this, the addition of H_2_O_2_ could improve ROS levels and save the inhibitory effect of OMT on osteoclast differentiation and SREBP2 activation. Thus, RANKL stimulation contributes to the production of ROS, which induces the activation of SREBP2. This pathway may be an important regulator in the early process of osteoclast differentiation since we found that OMT inhibited osteoclast formation and activity mainly during the early stage of osteoclastogenesis *in vitro*. These results suggested that OMT suppressed osteoclast differentiation through inhibiting ROS/SREBP2/NFATc1 axis, which is a novel signaling axis that regulates osteoclast differentiation.

**FIGURE 7:**
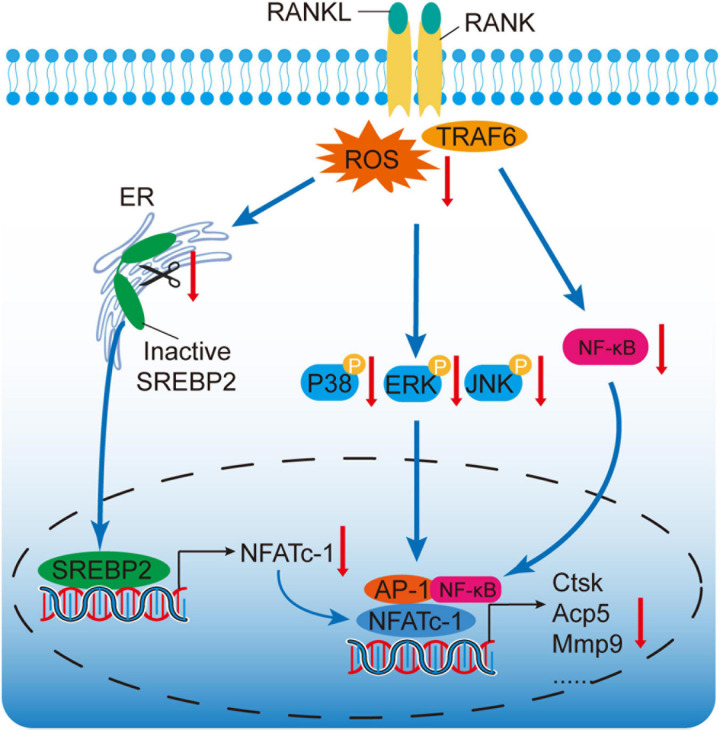
A proposed scheme for the mechanism by which OMT attenuates osteoclastogenesis via modulation of ROS-mediated SREBP2 Signaling. RANKL stimulation could induce ROS generation and activate downstream MAPK and NF-κB pathways. Active-SREBP2 transcriptionally controls the expression of NFATc1 and promotes osteoclastogenesis. OMT, a quinazine alkaloid, suppresses ROS levels, which attenuates the activation of SREBP2, MAPK, and NF-κB pathways, subsequently leading to attenuation of NFATc1 and osteoclastogenesis.

TRAF6, recruited by the binding of RANKL to RANK, is an important adaptor molecular which activates downstream MAPK and NF-κB pathways (Nakashima et al., 2012). Moreover, TRAF6 serves as an activator of ROS (Lee et al., 2005). It has been reported that a dominant-mutant form of TRAF6 attenuates the generation of intracellular ROS (Agidigbi and Kim, 2019). Our study indicated that OMT reduced ROS levels by down-regulating the degradation of TRAF6, thereby suppressing SREBP2 activation. Collectively, TRAF6/ROS/SREBP2/NFATc1 axis plays an important physiological function in osteoclastogenesis (Figure 7).

As an indispensable participant of bone homeostasis, osteoblast is important for bone formation (Chen et al., 2020). In the present study, we found no effect of OMT on osteoblast differentiation and mineralization *in vitro*. Based on these facts, an OVX mouse model was established to assess the potential therapeutic effects of OMT on osteoporosis. Consistent with the data *in vitro*, OMT could improve the bone loss induced by osteoporosis via attenuating osteoclastogenesis. Meanwhile, OMT treatment showed no effect on bone formation. These results indicates that OMT mainly serves as an anti-resorptive drug for osteoporosis. Although we did not observe significant toxicity in our *in vitro* as well as *in vivo* experiments, more safety tests are needed for future applications on osteoporosis.

In summary, this work has indicated for the first time that OMT could reduce ROS levels, which attenuates the activation of SREBP2, subsequently resulting in the attenuation of NFATc1 as well as its downstream osteoclast-specific genes. Additionally, OMT was also found to prevent OVX induced osteoporosis *in vivo*. Therefore, OMT may be a promising drug for the pharmacotherapy of osteoporosis in the future.

## Data Availability Statement

The raw data supporting the conclusions of this article will be made available by the authors, without undue reservation.

## Ethics Statement

The animal study was reviewed and approved by the National Institutes of Health (NIH) Guide for the Care and Use of Laboratory Animals and the guidelines for the animal treatment of Sir Run Run Shaw Hospital (Zhejiang University affiliated, Hangzhou, Zhejiang).

## Author Contributions

ZJ and CJ conceived and designed the experiments. CJ, SW, and QM performed the experiments. SW and QM conducted the animal study. ZJ, CJ, QM, AQ, and SF analyzed the data. AQ, SF, and ZJ supervised the experiments. CJ, QM, and ZJ drafted the manuscript. SF and AQ revised the manuscript. All authors approved the final version of the manuscript.

## Conflict of Interest

The authors declare that the research was conducted in the absence of any commercial or financial relationships that could be construed as a potential conflict of interest.

## Abbreviations

BMMs: bone marrow monocytes/macrophages
BV/TV: bone volume per tissue volume
CCK-8: Cell Counting Kit-8
Ctsk, dendritic cell-specific transmembrane protein: cathepsin K
Dc-stamp: dendrocyte expressed seven transmembrane protein
ERK: extracellular signal-regulated kinase
JNK: c-junN-terminal kinase
MAPKs: mitogen-activated protein kinases
M-CSF: Macrophage colony-stimulating factor
MMP: matrix metalloproteinase
NFATc1: nuclear factor of activated T cells 1
OMT: Oxymatrine
P38: p38 mitogen-activated protein kinases
qRT-PCR: Quantitative real-time polymerase chain reaction
RANKL: receptor activator of nuclear factor- κ B ligand
ROS: Reactive oxygen species
SREBP2: sterol regulatory element-binding protein 2
Tb.N: trabecular number
Tb.Sp: trabecular separation
Tb.Th: trabecular thickness
TRAF6: TNF receptor associated factor 6
Trap: tartrate-resistant alkaline phosphatase.
