# Corrigendum: Oxymatrine attenuates osteoclastogenesis via modulation of ROS-mediated SREBP2 signaling and counteracts ovariectomy-induced osteoporosis

**DOI:** 10.3389/fcell.2022.1002132

**Published:** 2022-09-09

**Authors:** Chao Jiang, Qingliang Ma, Shiyu Wang, Yang Shen, An Qin, Shunwu Fan, Zhiwei Jie

**Affiliations:** ^1^ Department of Orthopaedics, Sir Run Run Shaw Hospital, Zhejiang University School of Medicine, Hangzhou, China; ^2^ Key Laboratory of Musculoskeletal System Degeneration and Regeneration Translational Research of Zhejiang Province, Hangzhou, China; ^3^ Department of Orthopaedics, Shanghai Key Laboratory of Orthopaedic Implant, Shanghai Ninth People’s Hospital, Shanghai Jiao Tong University School of Medicine, Shanghai, China

**Keywords:** oxymatrine, osteoclast, ROS, SREBP2, osteoporosis

In the original article, there were mistakes in Figures 1, 3 as published. The scale bars of TRAP staining images in Figures 1D, 3J were incorrect. Furthermore, we applied a mismatched picture for Figure 1F. The corrected Figures 1, 3 appear below.

**FIGURE 1:**
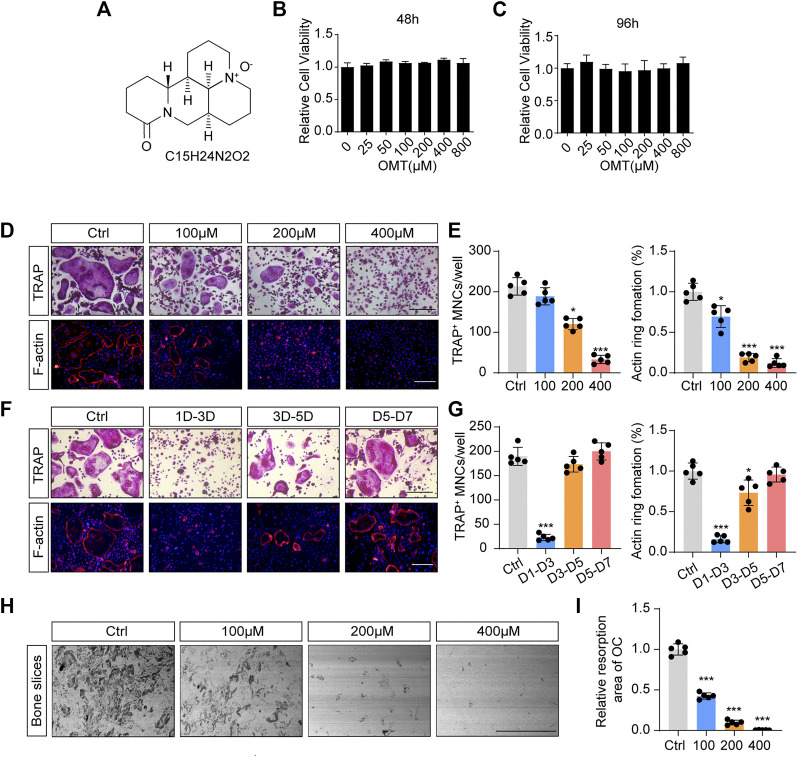
OMT inhibits RANKL-induced osteoclast formation and activity *in vitro*. **(A)** The chemical structure and formula of OMT. **(B,C)** Cell viability of OMT-treated BMMs at 48 and 96 h **(D)** BMMs were stimulated by 30 ng/mL M-CSF and 50 ng/mL RANKL, and treated with indicated concentrations of OMT for 5 days. Representative images of TRAP staining and F-actin staining were shown. Scale bar = 200 µm. **(E)** Quantification of TRAP-positive multinuclear cells and F-actin ring formation rate. **(F)** BMMs were stimulated with 30 ng/mL M-CSF and 50 ng/mL RANKL for 7 days, and treated with 200 µM OMT for the indicated days. TRAP staining and F-actin ring staining were performed. Scale bar = 200 µm. **(G)** Quantification of TRAP-positive multinuclear cells and F-actin ring formation rate. **(H)** Representative images of bone resorption pits. Scale bar = 500 µm. **(I)** Quantification of resorption pit area in each group. Data were presented as means ± SD of 5 independent experiments. *p < 0.05, ***p < 0.001.

**FIGURE 3:**
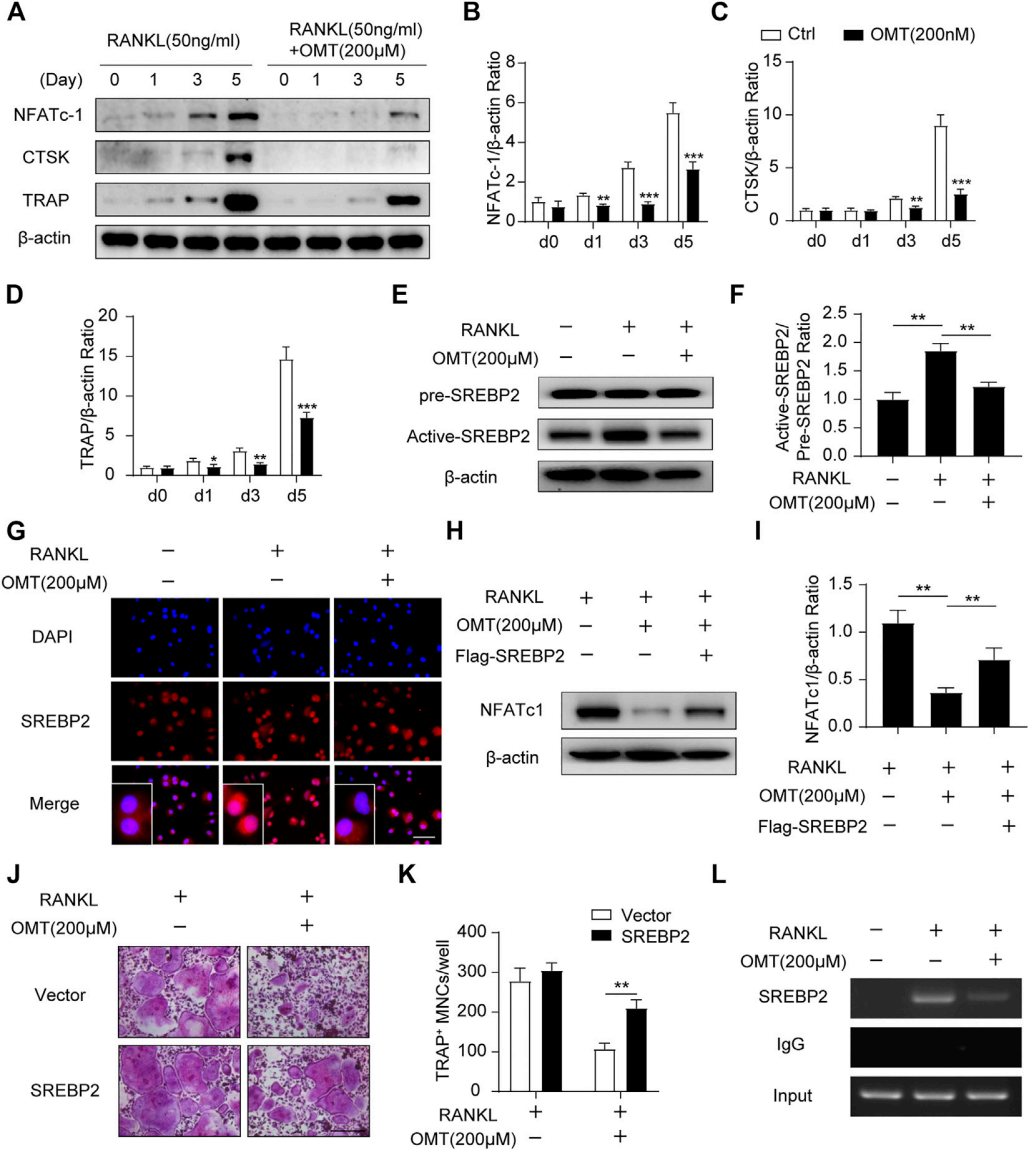
OMT attenuates SREBP2 activity and downstream NFATc1 expression during osteoclastogenesis. **(A)** BMMs were stimulated with RANKL, with or without 200 µM OMT for 0, 1, 3, 5 days, the expression of NFATc1, CTSK and TRAP was tested by western blots. **(B–D)** Quantification of the ratios of band intensity of NFATc1, TRAP, and CTSK relative to β-actin (*n* = 3 per group). **(E)** BMMs were treated with RANKL and 200 µM OMT as indicated, western blot was used to detect the level of pre-SREBP2 and active-SREBP2. **(F)** Quantification of active-SREBP2/pre-SREBP2 ratio (*n* = 3 per group). **(G)** RAW264.7 cells were treated with RANKL and OMT as indicated, immunofluorescence assay was performed to detect SREBP2 translocation. Scale bar = 100 µm. **(H)** BMMs were transfected with Flag-SREBP2 plasmid or empty vector, then treated with RANKL and OMT as indicated, the expression of NFATc1 was examined. **(I)** Quantification of NFATc1/β-actin ratio (n = 3 per group). **(J)** BMMs were transfected with Flag-SREBP2 plasmid or empty vector, then treated with RANKL and OMT as indicated. Representative images of TRAP staining were shown. Scale bar = 200 µm. **(K)** Quantification of TRAP-positive multinuclear cells per well (*n* = 5 per group). **(L)** ChIP assay was performed on BMMs, treated with RANKL and OMT as indicated. Data were presented as means ± SD. *p < 0.05, **p < 0.01, ***p < 0.001.

The authors apologize for these errors and state that this does not change the scientific conclusions of the article in any way. The original article has been updated.

