# Supplementary material for: Oxymatrine Attenuates Osteoclastogenesis via Modulation of ROS-Mediated SREBP2 Signaling and Counteracts Ovariectomy-Induced Osteoporosis
Source: Front Cell Dev Biol. 2021 May 31;9:684007. doi: 10.3389/fcell.2021.684007 (PMC8202524; doi:10.3389/fcell.2021.684007)
Supplement: Supplementary file 1 [file Data_Sheet_1.docx]

Supplementary Material

# Supplementary Figures


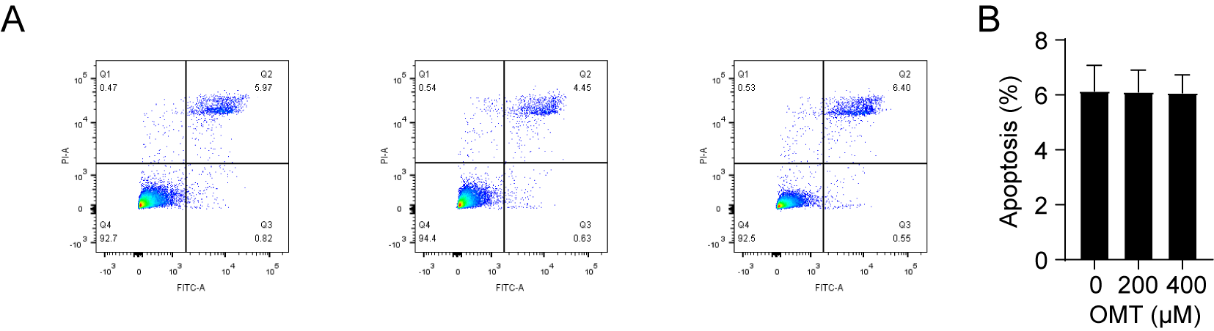


**Supplementary Figure 1. OMT has no effect on cell apoptosis.** (A) Representative images of cell apoptosis treated by OMT (0, 200, 400μM). (B) The percentage of apoptotic cells under OMT treatment.


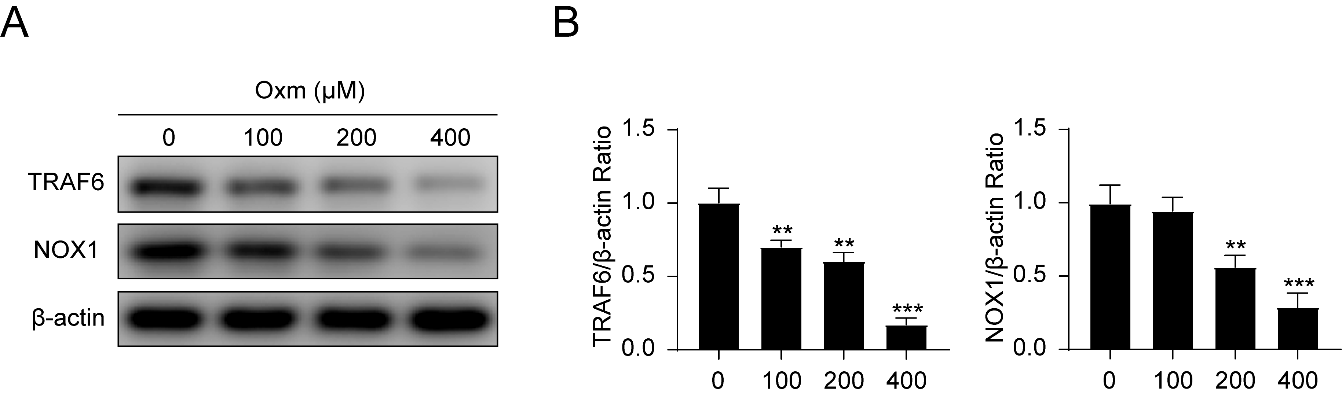


**Supplementary Figure 2.** **OMT inhibits protein expression of TRAF6 and NOX1.** (A) Representative Western Blot images of the effects of OMT on TRAF6 and NOX1. (B) Quantification of the ratios of band intensity of TRAF6 and NOX1 relative to β-actin (n=3 per group). Data were presented as means ± SD. **p*<0.05, ***p*<0.01, ****p*<0.001.


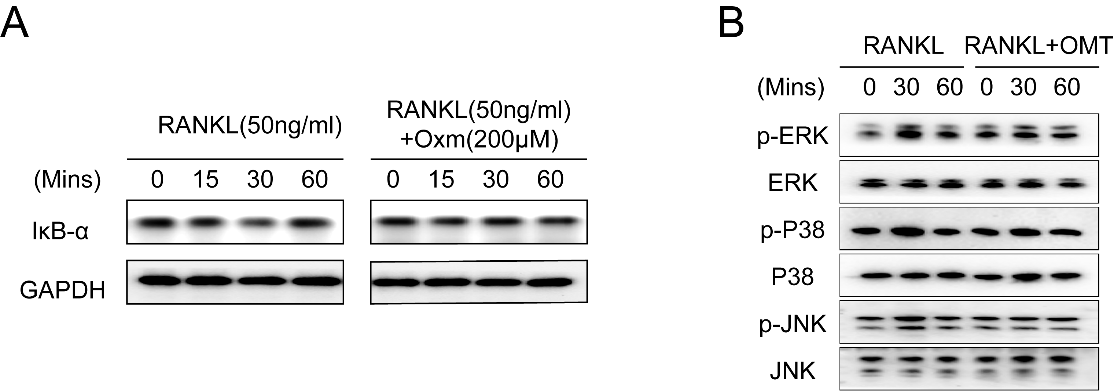


**Supplementary Figure 3.** **OMT inhibits NF-κB and MAPK pathways.** (A) Representative Western Blot images of the effects of OMT on IκBα degradation induced by RANKL. (B) Representative Western Blot images of the effects of OMT on MAPKs pathway, including p-ERK, p-P38, and p-JNK. All experiments were performed 3 times independently.


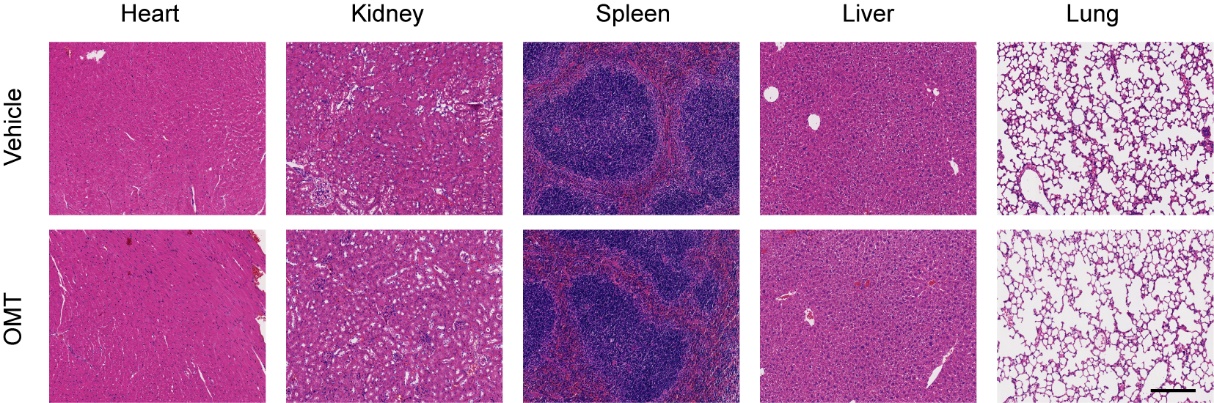


Supplementary Figure 4. OMT treatment didn’t affect major organs. H&E staining of the major organs from vehicle and OMT treatment groups. Scale bar=100μm.

# Supplementary Table

### Supplementary Table 1. Primer Sequences

| **Genes** | **Upstream (5′-3′)** | **Downstream (5′-3′)** |
| --- | --- | --- |
| *Gapdh* | ACCCAGAAGACTGTGGATG- | CACATTGGGGGTAGGAACAC |
| *Nfatc1* | CCGTTGCTTCCAGAAAATAACA | TGTGGGATGTGAACTCGGAA |
| *Trap* | CTGGAGTGCACGATGCCAGCGACA | TCCGTGCTCGGCGATGGACCAGA |
| *Dc-stamp* | AAAACCCTTGGGCTGTTCTT | AAT CATGGACGACTCCTTGG |
| *Ctsk* | CTTCCAATACGTGCAGCAGA | TCTTCAGGGCTTTCTCGTTC |
| *Mmp9* | AAAGGCAGCGTTAGCCAGAA | GTCCGTGAGGTTG GAGGTTT |
| *Atp6v0d2* | CAGAGCTGTACTTCAATGTGGAC | AGGTCTCACACTGCACTAGGT |
